# Supplementary material for: Promotion of Cyst Formation from a Renal Stem Cell Line Using Organ-Specific Extracellular Matrix Gel Format Culture System
Source: Gels. 2022 May 19;8(5):312. doi: 10.3390/gels8050312 (PMC9140708; doi:10.3390/gels8050312)
Supplement: Supplementary file 1 [file gels-08-00312-s001.zip › gels-1706455-supplementary.pdf]

gels

MDPI

Article

Promotion of Cyst Formation from a Renal Stem Cell Line Using Organ-Specific Extracellular Matrix Gel Format Culture System

Yusuke Sakai *, Yoshihiro Kubo, Nana Shirakigawa, Yoshinori Kawabe, Masamichi Kamihira and Hiroyuki Ijima *

Department of Chemical Engineering, Faculty of Engineering, Graduate School, Kyushu University, 744 Motooka, Nishi-ku, Fukuoka 819-0395, Japan; yoshi.mt09sp@gmail.com (Y.K.); nana.shirakigawa@kyudai.jp (N.S.); kawabe@chem-eng.kyushu-u.ac.jp (Y.K.); kamihira@chem-eng.kyushu-u.ac.jp (M.K.)

* Correspondence: y.sakai.bioeng@gmail.com (Y.S.); ijima@chem-eng.kyushu-u.ac.jp (H.I.)

Supplementary Information

Table S1. Mixing volume ratio for gel preparation.

Abbreviation	Collagen (3 mg/mL)	K- or L-ECM (9 mg/mL)	10x MEM	Reconstruction Buffer 1 (0.08 M NaOH, 0.2 M HEPES)	Reconstruction Buffer 2 (0.16 M NaOH, 0.2 M HEPES)
Collagen	8	-	1	1	-
K-ECM	6	2	1	-	1
L-ECM	6	2	1	-	1

Table S2. TaqMan gene expression assay numbers for real-time PCR analysis.

Gene Symbol	Gene Name	TaqMan Assay No.
T	*T brachyury transcription factor*	Cg04449691_g1
Osr1	*Odd-skipped related transcription factor 1*	Cg04509274_mH
Pax8	*Paired box 8*	Cg00440626_m1
Aqp1	*Aquaporin 1*	Cg04532077_m1
Slc5a1	*Solute carrier family 5, member 1*	Cg04551404_m1
Kcnj1	*Potassium inwardly-rectifying channel, subfamily J, member 1*	Cg04642736_s1
Gata3	*GATA binding protein 3*	Cg04569191_m1
Aqp2	*Aquaporin 2*	Cg04429866_m1
Gapdh	*Glyceraldehyde-3-phosphate dehydrogenase*	Cg04424038_gH

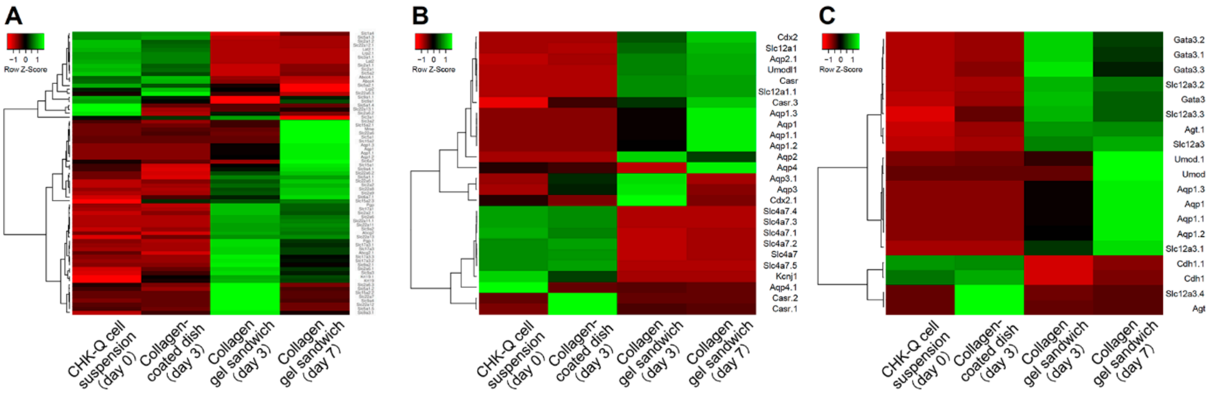

Figure S1. Gene expression heat map of (**A**) proximal tubule, (**B**) intermediate tubule, and (**C**) distal tubule in the uriniferous tubule. Pearson correlation was selected as the distance metric; average linkage clustering was selected as the linkage method.
